# Correction: Catalyst: Fast and flexible modeling of reaction networks

**DOI:** 10.1371/journal.pcbi.1013175

**Published:** 2025-06-12

**Authors:** Torkel E. Loman, Yingbo Ma, Vasily Ilin, Shashi Gowda, Niklas Korsbo, Nikhil Yewale, Chris Rackauckas, Samuel A. Isaacson

In Fig 3 of [1], Fig 3f is erroneously duplicated as Fig 3g, due to an error in the code used to prepare the figure. With this Correction, the first and corresponding author TEL provides a revised Fig 3 with the correct panel for Fig 3g.

**Fig 3:**
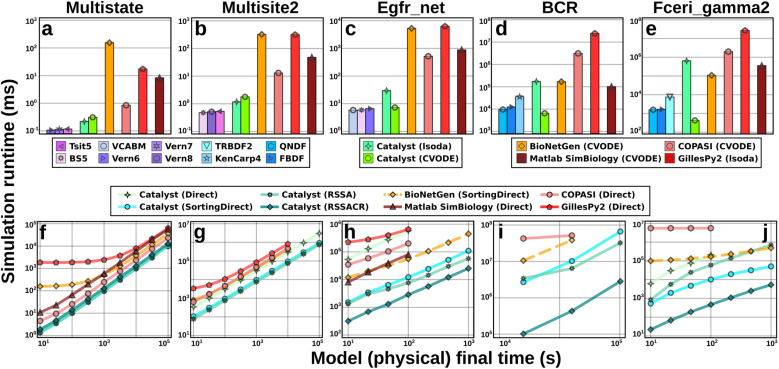
Simulations of Catalyst models outperform those of other modeling packages. Benchmarks of simulation runtimes for Catalyst and four other modeling packages (BioNetGen, COPASI, GillesPy2, and Matlab SimBiology). The benchmarks were run on the multi-state (Multistate, 9 species and 18 reactions [47]), multi-site (Multisite 2, 66 species and 288 reactions [48]), epidermal growth factor receptor signalling (Egfr_net, 356 species and 3749 reactions [49]), B-cell receptor (1122 species and 24388 reactions [50]), and high-affinity human IgE receptor signalling (Fceri_gamma2, 3744 species and 58276 reactions [51]) models. (a-e) Benchmarks of deterministic RRE ODE simulations of the five models. Each bar shows, for a given method, the runtime to simulate the model (to steady-state for those that approach a steady-state). For Catalyst, we show the three best-performing native Julia methods, as well as the performance of lsoda and CVODE. For each of the other tools, we show its best-performing method. Identical values for absolute and relative tolerance are used across all packages and methods. For each benchmark, the method options used can be found in Section 4.1, the exact benchmark times in Table A in S1 Text, and further details on the solver options for each tool in Section B in S1 Text. While this figure only contains the most performant methods, a full list of methods investigated can be found in Section B in S1 Text, with their results described in Figs A and B in S1 Text. (f-j) Benchmarks of stochastic chemical kinetics SSA simulations of the five models. Via JumpProcesses.jl, Catalyst can use several different algorithms (e.g., Direct, Sorting Direct, RSSA, and RSSACR above) for exact Gillespie simulations. Here, the simulation runtime is plotted against the (physical) final time of the simulation. Due to their long runtimes, some tools were not benchmarked for the largest models. We note that, in [52], it was remarked that BioNetGen (dashed orange lines) use a pseudo-random number generator in SSAs that, while fast, is of lower quality than many (slower) modern generators such as Mersenne Twister. For full details on benchmarks, see Section 4.1.

The first and corresponding author has also added a branch to the repository hosting the corrected scripts for generating all figures presented in [1], as well as for carrying out the benchmarks. This additional branch, titled, “duplication_fix”, includes the corrected figure used in Fig 3, and the final version of all figures presented in [1]. It is available from the following URL: https://github.com/SciML/Catalyst_PLOS_COMPBIO_2023/tree/duplication_fix.

The authors apologize for the error in the published article.

## References

[1] Loman TE, Ma Y, Ilin V, Gowda S, Korsbo N, Yewale N, et al. Catalyst: Fast and flexible modeling of reaction networks. PLoS Comput Biol. 2023;19(10):e1011530. doi: 10.1371/journal.pcbi.1011530 37851697 PMC10584191

