# Supplementary material for: Catalyst: Fast and flexible modeling of reaction networks
Source: PLoS Comput Biol. 2023 Oct 18;19(10):e1011530. doi: 10.1371/journal.pcbi.1011530 (PMC10584191; doi:10.1371/journal.pcbi.1011530)
Supplement: S1 Text — (PDF) [file pcbi.1011530.s001.pdf]

Catalyst: Fast and flexible modeling of reaction networks - S1 Text - Additional benchmarks and benchmark information

A Exact ODE benchmark times

This section contains Table A in S1 Text, describing the exact benchmark times plotted in Figure 3 A-E.

Table A. ODE Benchmark times.

Model:	**Multistate**	**Multisite2**	**Egfr_net**	**BCR**	**Fceri_gamma2**
Julia solver 1	0.107	0.477	5.89	9,550	1,570
Julia solver 2	0.114	0.517	5.91	12,500	1,670
Julia solver 3	0.117	0.535	6.65	35,900	7,510
Catalyst lsoda	0.220	1.17	29.7	172,000	644,000
Catalyst CVODE	0.310	1.78	7.31	6,520	423
BioNetGen	154	322	5,280	170,000	108,000
COPASI	0.849	13.0	520	3,110,000	1,990,000
GillesPy2	17.2	315	6,280	24,400,000	26,800,000
Matlab	8.34	47.8	880	101,000	354,000

Main text Fig 3 A-E shows the results as bar charts, here, the same benchmark times (median over several simulations) are given as numbers (in units of milliseconds). Each field corresponds to the same field in Main text Table 3.

B List of ODE benchmarks

Here follows a list of all ODE simulation methods, and combinations of options, used for each tool. The best-performing combinations are shown in Fig 3 (with details of the used options listed in Table 3). The results of all combinations listed here can be found in Figs A and B in S1 Text. For some settings, a parenthesis further clarifies what the option implies. For all ODE simulations, the absolute tolerance was set to `1e-9` and the relative tolerance to `1e-6`.

B.1 BioNetGen solvers

The `ode` method is the CVODE solver, by default using dense LU and a finite difference Jacobian approximation. The `n_steps` parameter sets the number of saved steps. We sat this to 1, to ensure no intermediary time points were saved. Setting `sparse = true` enables the (un-preconditioned) GMRES linear solver (for which we used the default GMRES tolerance). BioNetGen does not seem to offer an option to specify a preconditioner for GMRES.

- `Method` = `ode`, `n_steps` = `1`, `sparse` = `false`.
- `Method` = `ode`, `n_steps` = `1`, `sparse` = `true`.

B.2 Catalyst solvers

The `CVODE_BDF` sovler is the CVODE solver. The `KrylovJL_GMRES` linear solver is the GMRES linear solver (for which we used the default GMRES tolerance). The `jac` = `true` options set that a symbolic Jacobian is built, else, the Jacobian is computed through automatic differentiation (or if `autodiff` = `false` is used, through finite differences). If the `sparse` = `true` is used, a sparse Jacobian representation is used.

- Solver = `lsoda`.
- Solver = `CVODE_BDF`.
- Solver = `CVODE_BDF`, `linear_solver` = `LapackDense`.
- Solver = `CVODE_BDF`, `linear_solver` = `GMRES`.
- Solver = `CVODE_BDF`, `linear_solver` = `GMRES`, `sparse` = `true`, iLU preconditioner.
- Solver = `CVODE_BDF`, `linear_solver` = `KLU`, `sparse` = `true`, `jac` = `true`.
- Solver = `TRBDF2`.
- Solver = `TRBDF2`, `linsolve` = `KrylovJL_GMRES`.
- Solver = `TRBDF2`, `linsolve` = `KrylovJL_GMRES`, `sparse` = `true`, iLU preconditioner.
- Solver = `TRBDF2`, `linsolve` = `KLUFactorization`, `sparse` = `true`, `jac` = `true`.
- Solver = `KenCarp4`.
- Solver = `KenCarp4`, `linsolve` = `KrylovJL_GMRES`.
- Solver = `KenCarp4`, `linsolve` = `KrylovJL_GMRES`, `sparse` = `true`, iLU preconditioner.

- Solver = `KenCarp4`, `linsolve` = `KLUFactorization`, `sparse` = `true`, `jac` = `true`.
- Solver = `QNDF`.
- Solver = `QNDF`, `linsolve` = `KrylovJL_GMRES`.
- Solver = `QNDF`, `linsolve` = `KrylovJL_GMRES`, `sparse` = `true`, iLU preconditioner.
- Solver = `QNDF`, `linsolve` = `KLUFactorization`, `sparse` = `true`, `jac` = `true`.
- Solver = `FBDF`.
- Solver = `FBDF`, `linsolve` = `KrylovJL_GMRES`.
- Solver = `FBDF`, `linsolve` = `KrylovJL_GMRES`, `sparse` = `true`, iLU preconditioner.
- Solver = `FBDF`, `linsolve` = `KLUFactorization`, `sparse` = `true`, `jac` = `true`.
- Solver = `Rodas4`.
- Solver = `Rodas4`, `linsolve` = `KrylovJL_GMRES`.
- Solver = `Rodas4`, `linsolve` = `KrylovJL_GMRES`, `sparse` = `true`, iLU preconditioner.
- Solver = `Rodas4`, `linsolve` = `KLUFactorization`, `sparse` = `true`, `jac` = `true`.
- Solver = `Rodas5P`.
- Solver = `Rodas5P`, `linsolve` = `KrylovJL_GMRES`.
- Solver = `Rodas5P`, `linsolve` = `KrylovJL_GMRES`, `sparse` = `true`, iLU preconditioner.
- Solver = `Rodas5P`, `linsolve` = `KLUFactorization`, `sparse` = `true`, `jac` = `true`.
- Solver = `Rosenbrock23`.
- Solver = `Rosenbrock23`, `linsolve` = `KrylovJL_GMRES`.
- Solver = `Rosenbrock23`, `linsolve` = `KrylovJL_GMRES`, `sparse` = `true`, iLU preconditioner.
- Solver = `Rosenbrock23`, `linsolve` = `KLUFactorization`, `sparse` = `true`, `jac` = `true`.
- Solver = `Tsit5`.
- Solver = `BS5`.
- Solver = `VCABM`.
- Solver = `Vern6`.
- Solver = `Vern7`.
- Solver = `Vern8`.
- Solver = `Vern9`.

- Solver = `ROCK2`.
- Solver = `ROCK4`.

The iLU preconditioner option simulations were benchmarked only for the two largest models (BCR and Fceri_gamma2). The explicit solvers (`Tsit5`, `BS5`, `VCABM`, `Vern6`, `Vern7`, `Vern8`, `Vern9`, `ROCK2`, and `ROCK4`) were not benchmarked for the BCR model.

For all benchmarks, we used the `saveat` = [Simulation length] option to ensure no intermediary time points were saved. For the `TRBDF2`, `KenCarp4`, `QNDF`, `FBDF`, `Rodas4`, `Rodas5P`, `Rosenbrock23` methods, when benchmarked on the (large) BCR and Fceri_gamma2 models, the `autodiff` = `false` option was used to disable automatic differentiation. The iLU preconditioner requires setting a single parameter τ. For CVODE we used $\tau = 5$ (BCR model) and $\tau = 1e2$ (Fceri_gamma2 model). For the other methods, we used $\tau = 1e12$ (BCR model) and $\tau = 1e2$ (Fceri_gamma2 model). While these τ values were roughly optimized for their specific problems, we note that for both the BCR and Fceri_gamma2 models, the fastest Catalyst solvers included solvers not depending on preconditioners with customized τ values.

B.3 COPASI solvers

The `stepsize` option sets the time between saving the solution. We used the length of the simulation to ensure no intermediary time points were saved.

- `method` = `deterministic` (CVODE), `stepsize` = [Simulation length].

B.4 GillesPy2 solvers

The `nsteps` option sets the maximum number of time steps. The `increment` option sets the time between saving the solution. We used the length of the simulation to ensure no intermediary time points were saved. The `solver` = `ODESolver` options designate that we run ODE simulations (as opposed to e.g. Gillespie simulations), while the `integrator` sets the numerical solver method used (e.g. lsoda).

- `solver` = `ODESolver`, `integrator` = `lsoda`, `nsteps` = `100000`, `increment` = [Simulation length].
- `solver` = `ODESolver`, `integrator` = `csolver`, `nsteps` = `100000`, `increment` = [Simulation length].
- `solver` = `ODESolver`, `integrator` = `zvode`, `nsteps` = `100000`, `increment` = [Simulation length].
- `solver` = `ODESolver`, `integrator` = `vode`, `nsteps` = `100000`, `increment` = [Simulation length].

Due to their poor performance compared to `lsoda`, the `zvode` and `vode` methods benchmarks were investigated beyond initial tests. Furthermore, due to its poor performance, the `csolver` solver was not benchmarked for the two largest (BCR and Fceri_gamma2) models.

B.5 Matlab solvers

The `sundials` solver type is the CVODE solver, using its default options. For Matlab there appeared to be no other documented control parameters (including setting the density at which time points were saved) that could beneficially affect the performance. We tried the `RuntimeOptions.StatesToLog = {}` option, however, this offered no improvement, while reducing performance for the smaller models.

- `SolverType` = `sundials`.

C List of SSA benchmarks

Here follows a list of all SSA simulation methods used for each tool. Their performance is shown in Fig 3 (due to the small number of SSA methods, all fit in Fig 3 and no supplementary figure was needed).

C.1 BioNetGen solvers

The `ssa` method is the Sorting Direct method. The `n_steps` parameter sets the number of saved steps. We sat this to 1, to ensure no intermediary time points were saved.

- `method` = `ssa`, `n_steps` = `1`.

Due to the benchmarks surpassing the 4-day limit of jobs at the used HPC supercomputer, the `ssa` method was not benchmarked on the BCR model.

C.2 Catalyst solvers

Here, the `save_positions` = `(false,false)` options prevent the saving of the solution before/after a jump is made (which will severely reduce performance when a large number of jumps are performed). Using this option, we ensured that no intermediary time points were saved.

- Solver = `Direct`, `save_positions` = `(false,false)`
- Solver = `SortingDirect`, `save_positions` = `(false,false)`
- Solver = `RSSA`, `save_positions` = `(false,false)`
- Solver = `RSSACR`, `save_positions` = `(false,false)`

C.3 Copasi solvers

The `stepsize` option sets the time between saving the solution. We used the length of the simulation to ensure no intermediary time points were saved.

- `method` = `directMethod`, `stepsize` = [Simulation length].

Due to the benchmarks surpassing the 4-day limit of jobs at the used HPC supercomputer, COPASI was not benchmarked on the BCR model.

C.4 GillesPy2 solvers

The `nsteps` option sets the maximum number of time steps. The `increment` option sets the time between saving the solution. We used the length of the simulation to ensure no intermediary time points were saved.

- `solver` = `ssa`, `increment` = [Simulation length].
- `solver` = `numpyssa`, `increment` = [Simulation length].

Due to its poor performance compared to `ssa`, the `numpyssa` method benchmarks were never concluded beyond initial tests. Due to the benchmarks surpassing the 4-day limit of jobs at the used HPC supercomputer, the `ssa` method was not benchmarked on the BCR and Fceri_gamma2 models.

C.5 Matlab solvers

The `ssa` solver type is Gillespie's Direct method. For Matlab there appeared to be no other documented control parameters (including setting the density at which time points were saved) that could affect the performance.

- `SolverType` = `ssa`.

Due to slow performance and/or crashing for larger models, the `ssa` method was not benchmarked on the BCR and Fceri_gamma2 models.

D Additional benchmarks of ODE solvers

In this article we compare ODE simulation benchmarks for Catalyst and various other CRN modelling tools (BioNetGen, COPASI, GillesPy2, and Matlab's SimBiology toolbox) (Fig 3). While we benchmarked a large number of combinations of methods and tools (S1 Text Section B), the main text figure only displays the results using the best combinations. Here, in Figs A and B in S1 Text, we show the benchmarks for the full set. Fig A in S1 Text shows the run time to reach each models' steady states (or, for the BCR model, complete 3 pulses), while Fig B in S1 Text shows the simulation times as a function of the real (physical) end time of the simulation. The exact options used for each simulation are described in S1 Text Section B.

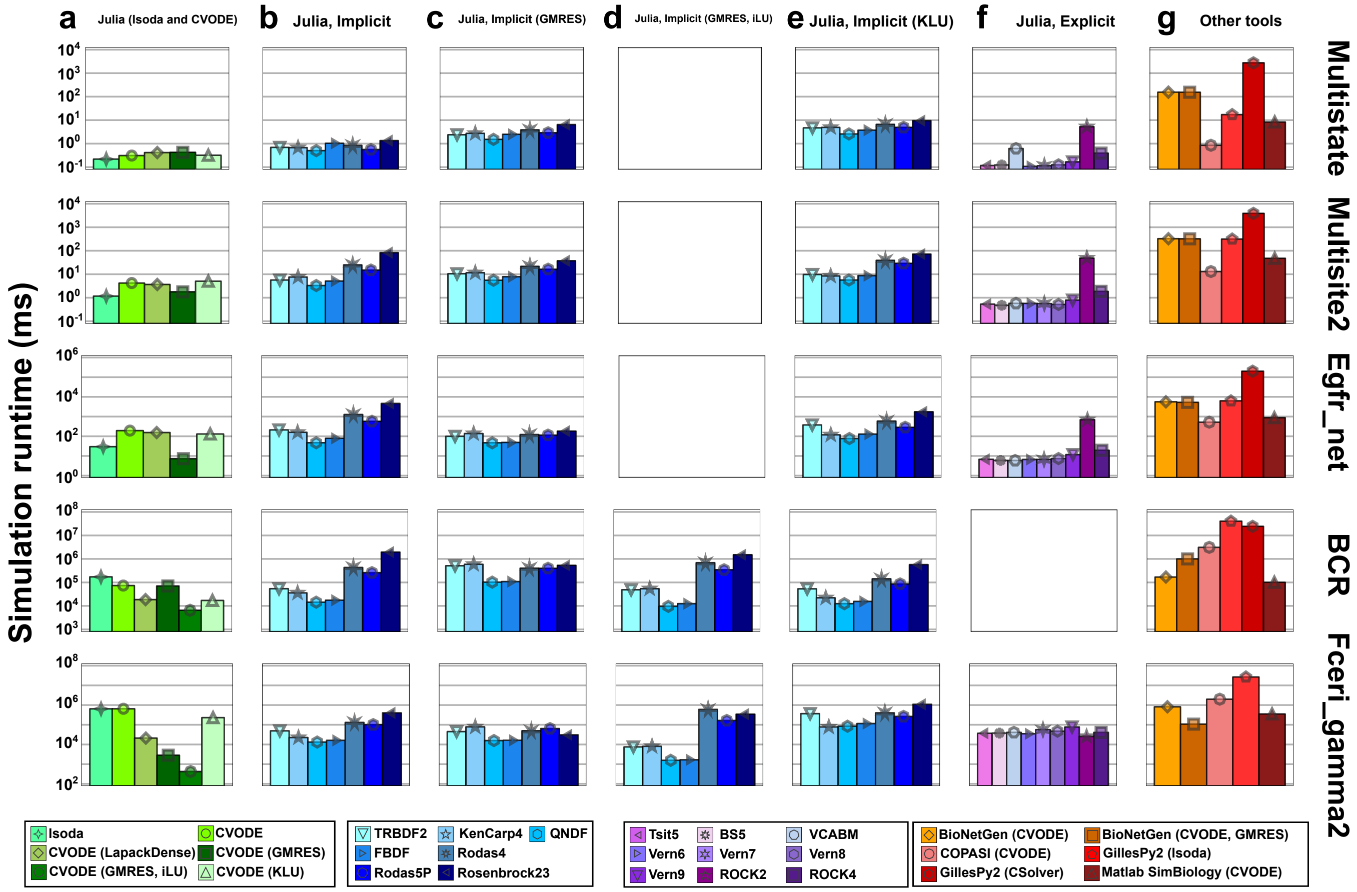

Fig A. The result of the benchmarks across the full set of ODE solvers and options. These benchmarks are a more extensive set than what is provided in Fig 3 (which includes only the best alternative for each combination of tool and model). (a) Benchmarks for Catalyst, using lsoda, as well as CVODE with a range of options (no linear solver specified, or for CVODE, the LapackDense, GMRES, or KLU linear solvers). The GMRES option was trialed with and without the iLU preconditioner. A sparse Jacobian representation was used when the KLU linear solver or iLU preconditioner was used. Furthermore, for the KLU linear solver a symbolic, sparse, Jacobian was used. (b) Catalyst benchmarks using an extended set of (implicit) ODE solvers. (c) Benchmark for the same solvers as in B, but with the GMRES linear solver. (d) Same as in C, but using an iLU preconditioner and sparse finite-difference Jacobian representation. (e) Benchmark for the same solvers as in B, but with the KLU linear solver and sparse, symbolic, Jacobian representation. (f) Catalyst benchmarks using an extended set of (explicit) ODE solvers. (g) Benchmarks using non-Catalyst tools. For more details on the options used for each benchmark, please see S1 Text Section B.

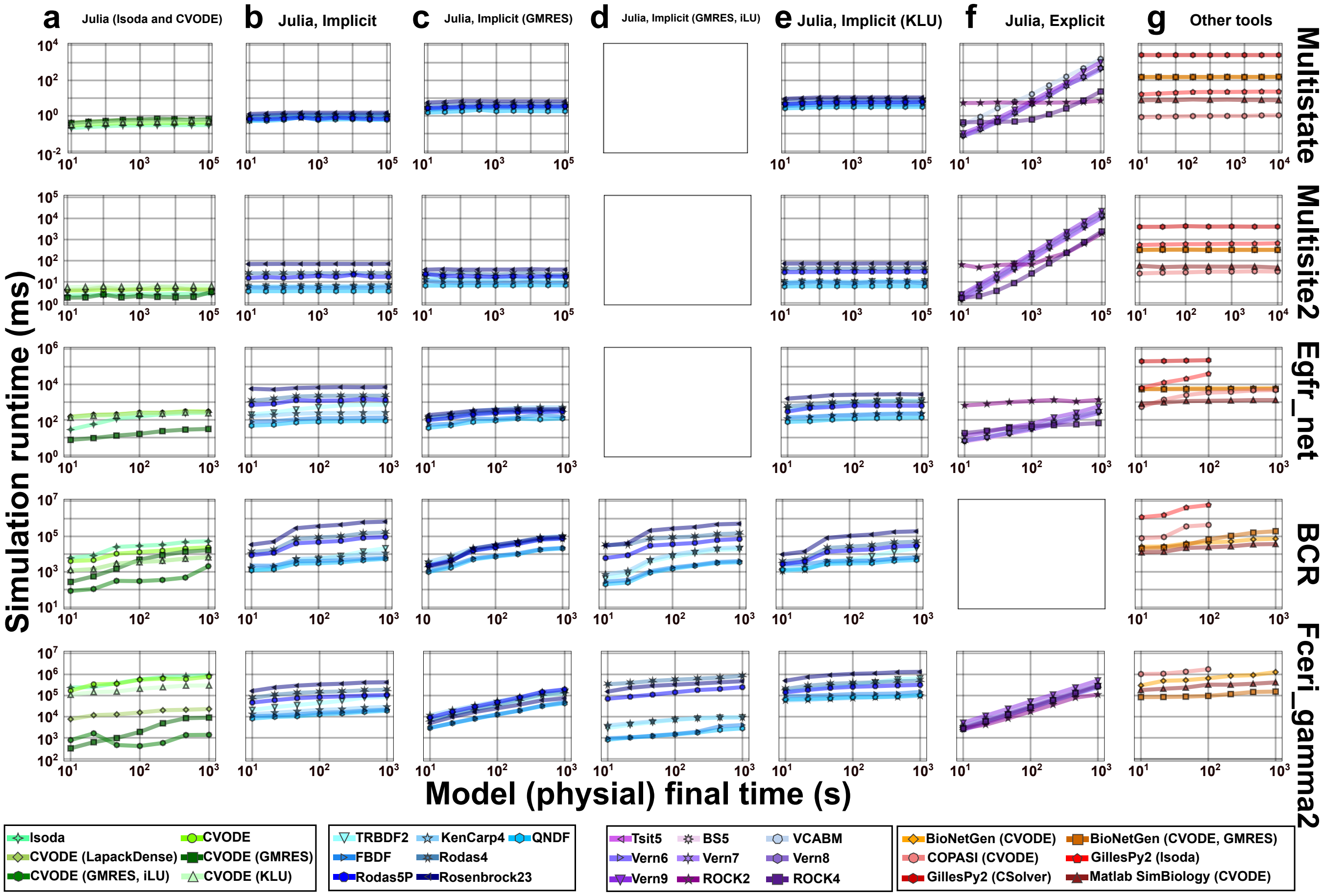

Fig B. The result of the benchmarks across the full set of ODE solvers and options. These benchmarks are a more extensive set than what is provided in Fig 3 (which includes only the best alternative for each combination of tool and model). Here, the simulation run time was as a function of the real (physical) end time of the simulation. (a) Benchmarks for Catalyst, using lsoda, as well as CVODE with a range of options (no linear solver specified, or for CVODE, the LapackDense, GMRES, or KLU linear solvers). The GMRES option was trialed using with and without the iLU precondition. A sparse Jacobian representation was used when the KLU linear solver or iLU preconditioner was used. Furthermore, for KLU linear solver, a symbolic, sparse, Jacobian was used. (b) Catalyst benchmarks using an extended set of (implicit) ODE solvers. (c) Benchmark for the same solvers as in B, but with the GMRES linear solver. (d) Same as in C, but using an iLU preconditioner and a sparse finite-difference Jacobian representation. (e) Benchmark for the same solvers as in B, but with the KLU linear solver and sparse, symbolic, Jacobian representation. (f) Catalyst benchmarks using an extended set of (explicit) ODE solvers. (g) Benchmarks using non-Catalyst tools. For more details on the options used for each benchmark, please see S1 Text Section B.

E Work-precision diagrams for Julia solvers

For the best-performing Julia solvers, we compare the run time of the numerical simulator to the error it generates. For each combination of solver and options, we make repeated simulations at various tolerances (absolute and real tolerance both equal to 10^{-5}, 10^{-6}, 10^{-7}, or 10^{-8}). For each simulation, we calculate the error by comparison to a single simulation using the CVODE solver and no options, with absolute and real tolerances for the latter equal to 10^{-12}. For each tolerance, we can thus compute the mean error and simulation time (across all simulations at that tolerance). Plotting these, we generate a so-called work-precision diagram. Such diagrams for all models are shown in Fig C in S1 Text.

Unlike all other benchmarks, the work-precision diagrams were not computed on supercloud, but rather on the SciML organization's benchmarking server, which has the following specifications:

OS Linux (x86_64-linux-gnu)

CPU 128 × AMD EPYC 7502 32-Core Processor

WORD_SIZE 64

LIBM libopenlibm

LLVM libLLVM-13.0.1 (ORCJIT, znver2)

Threads 128 on 128 virtual cores

The code that was used to generate Fig C in S1 Text can be found in the associated Github benchmarking repository for the article, see main text Section 6.2. For a more extensive set of work-precision diagrams of available Julia (and thus Catalyst) solvers, please refer to the SciMLBenchmarks.jl package.

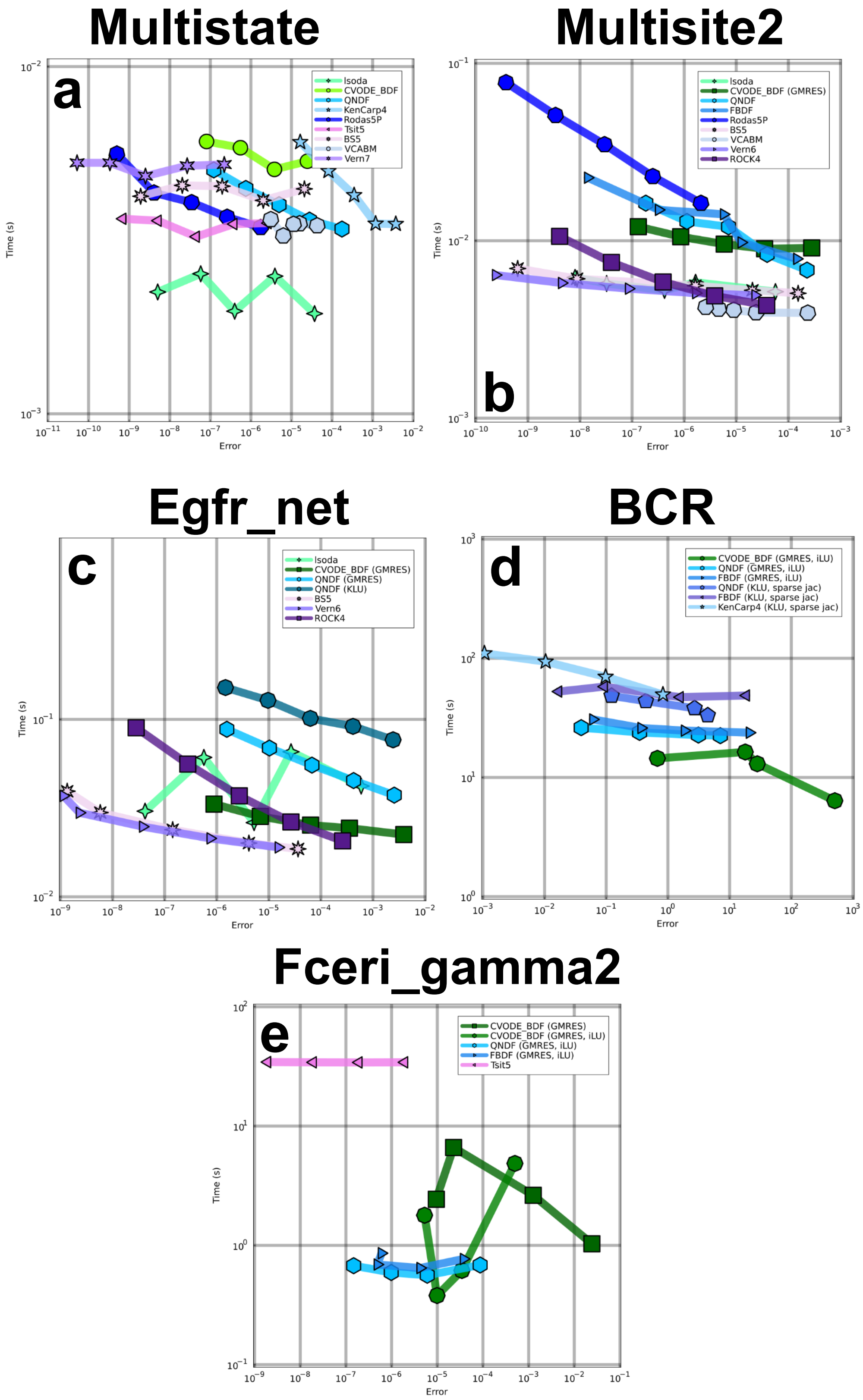

Fig C. Work-precision diagrams for selected Julia ODE solvers (and options). Native Julia solvers typically have smaller errors (compared to lsoda and CVODE) when tolerances are kept identical. The simulation options used for this figure are drawn from the list in S1 Text Section B.

F Simulation trajectories for various modelling tools

In this article, we compare ODE and SSA simulation benchmarks for Catalyst and various other CRN modelling tools (BioNetGen, COPASI, GillesPy2, and Matlab's SimBiology toolbox). To ensure that each tool successfully simulates each model, we here plot the output trajectories for each combination of tool and model. These plots are displayed in Figs D-M in S1 Text.

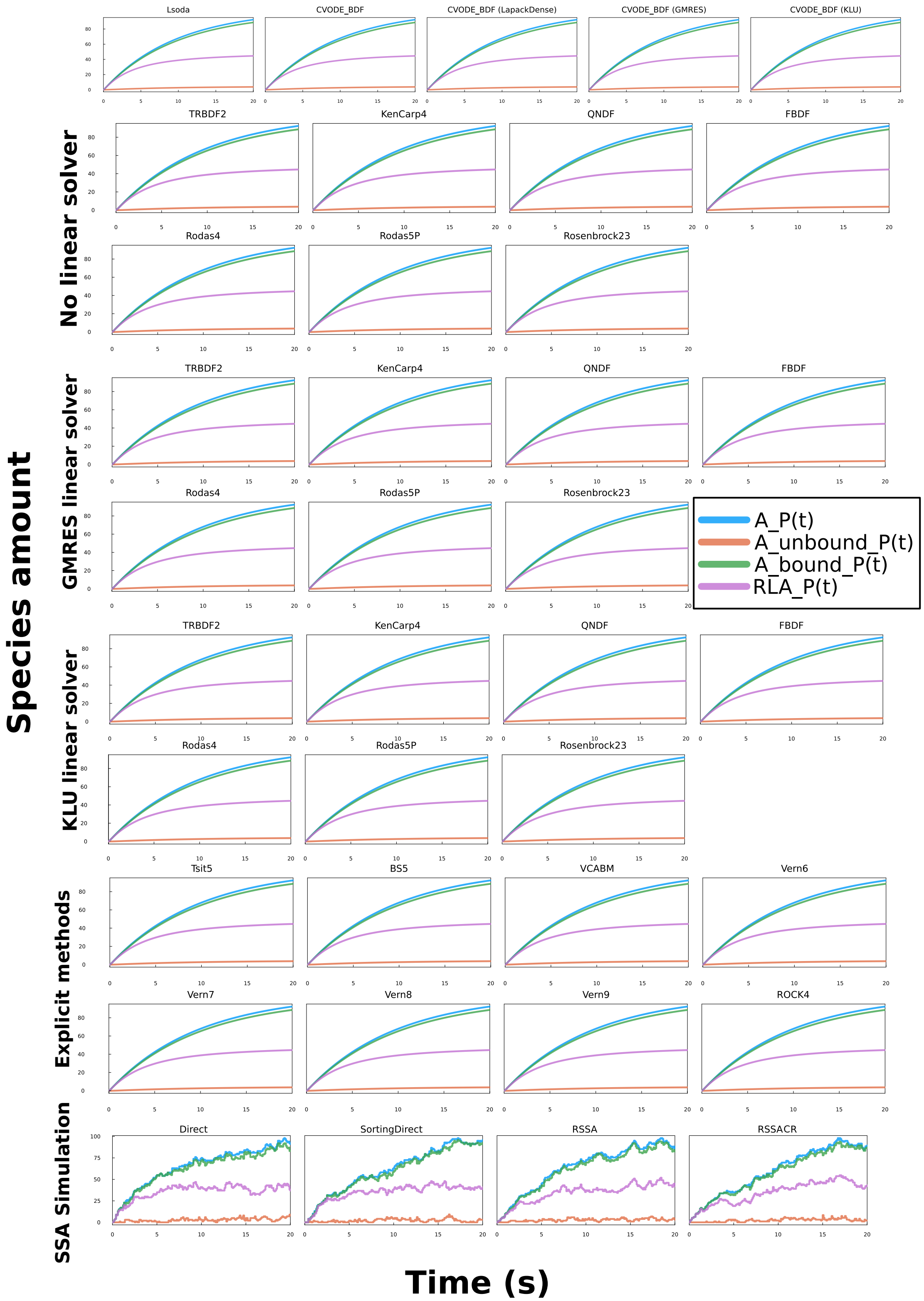

Fig D. Catalyst benchmark simulation time trajectories for the multistate model. The multistate model is simulated until it reaches its (approximate) steady state at $t = 20$ seconds (same time point which was used for the benchmarks in Fig 3). It was simulated using both ODE and SSA methods. The simulation trajectories correspond to those of the other tools, suggesting that the models are correctly interpreted.

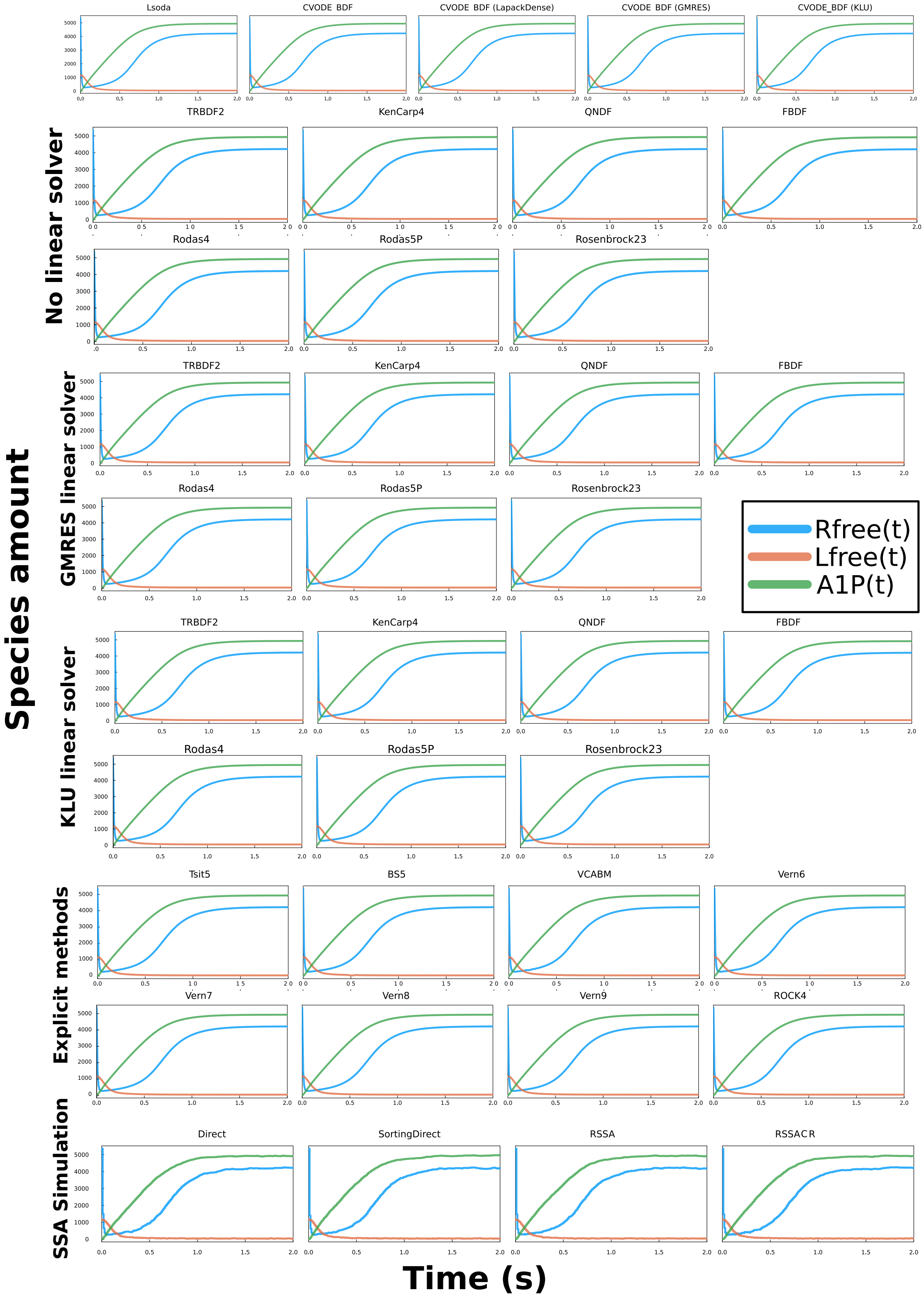

Fig E. Catalyst benchmark simulation time trajectories for the multisite2 model. The multisite2 model is simulated until it reaches its (approximate) steady state at $t = 2$ seconds (same time point which was used for the benchmarks in Fig 3). It was simulated using both ODE and SSA methods. The simulation trajectories correspond to those of the other tools, suggesting that the models are correctly interpreted.

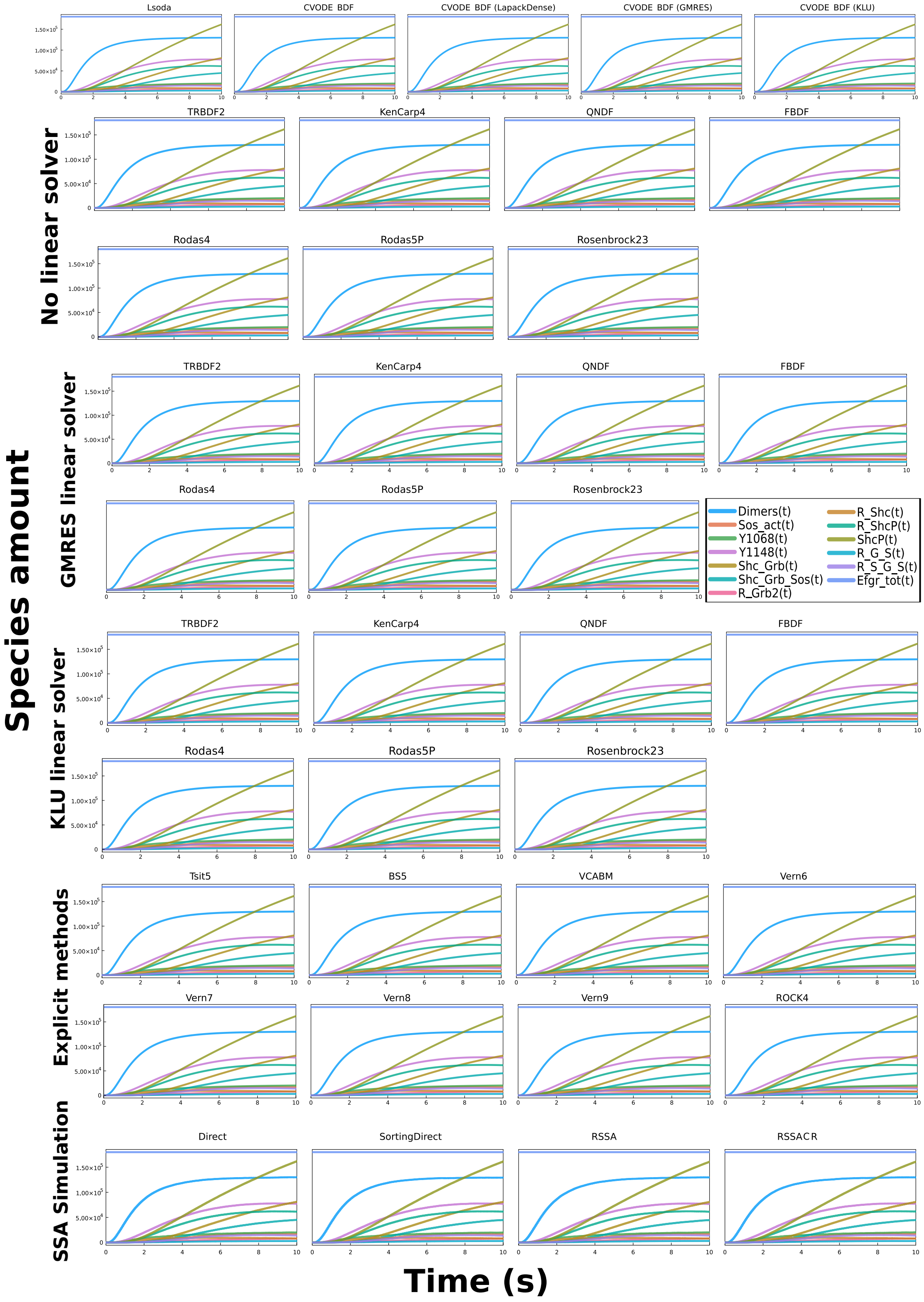

Fig F. Catalyst benchmark simulation time trajectories for the egfr_net model. The egfr_net model is simulated until it reaches its (approximate) steady state at $t = 10$ seconds (same time point which was used for the benchmarks in Fig 3). It was simulated using both ODE and SSA methods. The simulation trajectories correspond to those of the other tools, suggesting that the models are correctly interpreted.

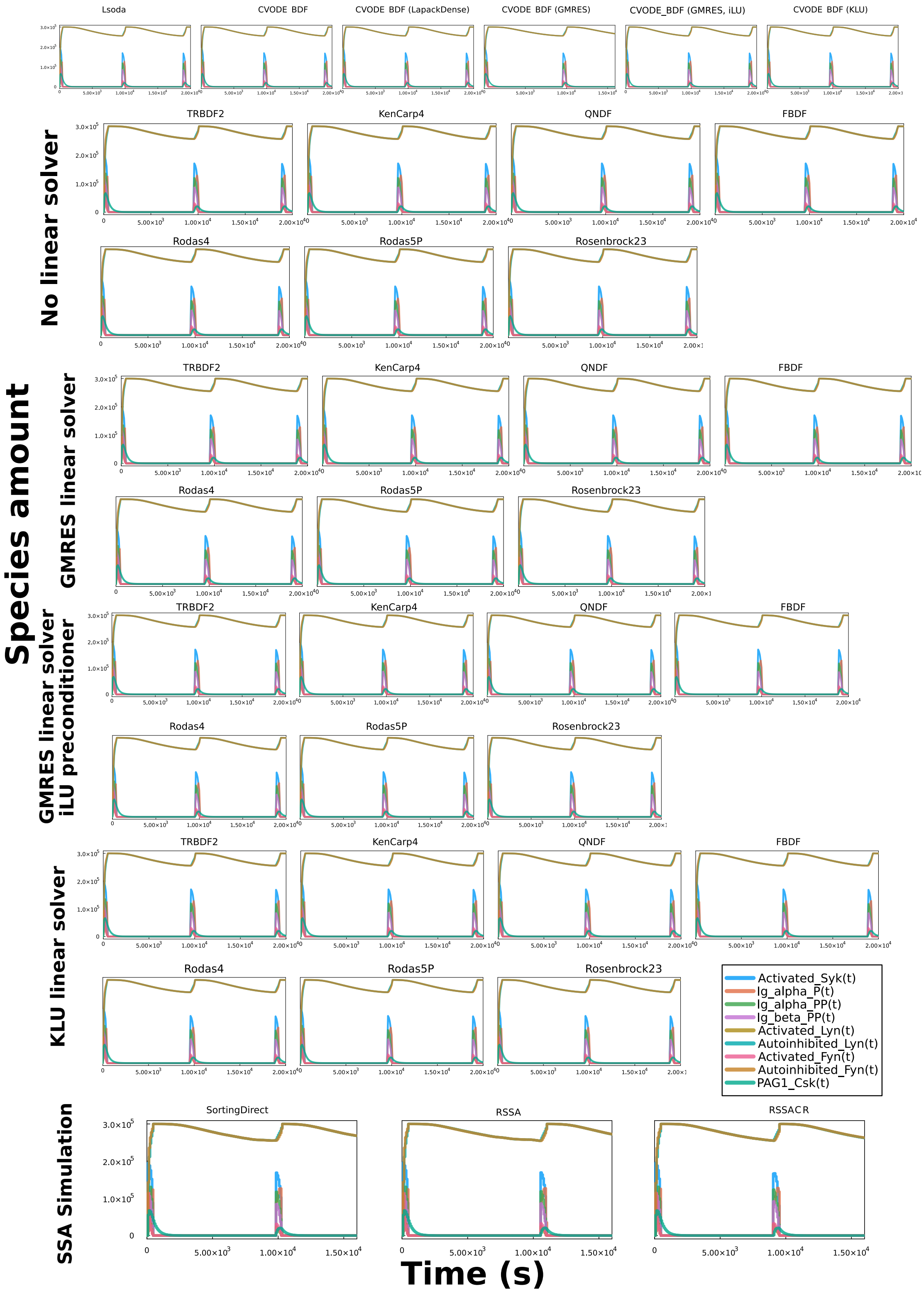

Fig G. Catalyst benchmark simulation time trajectories for the BCR model. The BCR model is simulated until it reaches its (approximate) steady state at $t = 10,000$ seconds (same time point which was used for the benchmarks in Fig 3). It was simulated using both ODE and SSA methods. The time of the SSA simulation's pulse initiation is variable, and hence the system was resimulated to ensure that a pulse was initiated in the simulation. The simulation trajectories correspond to those of the other tools, suggesting that the models are correctly interpreted.

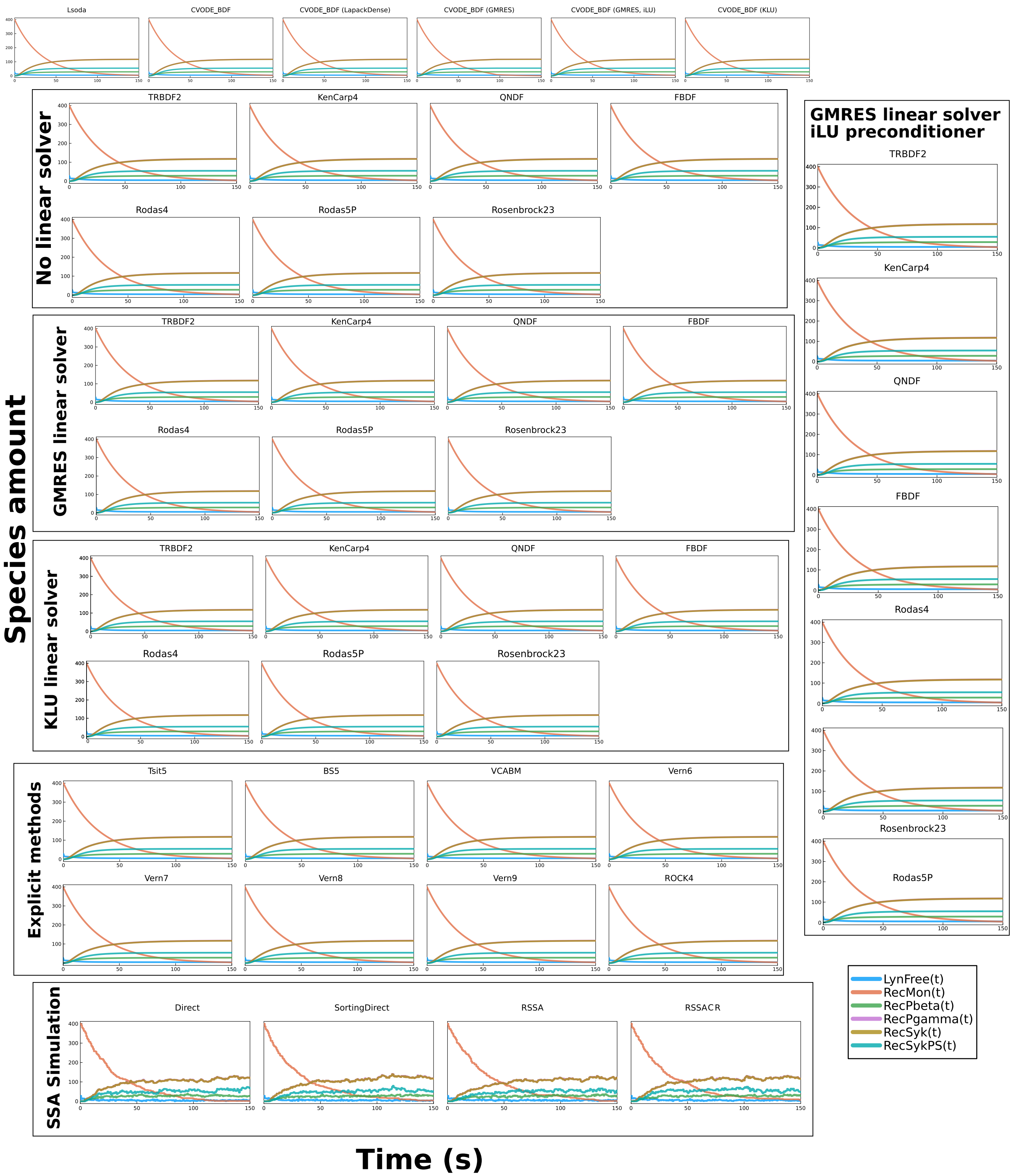

Fig H. Catalyst benchmark simulation time trajectories for the fceri_gamma2 model. The fceri_gamma2 model is simulated until it reaches its (approximate) steady state at $t = 150$ seconds (same time point which was used for the benchmarks in Fig 3). It was simulated using both ODE and SSA methods. The simulation trajectories correspond to those of the other tools, suggesting that the models are correctly interpreted.

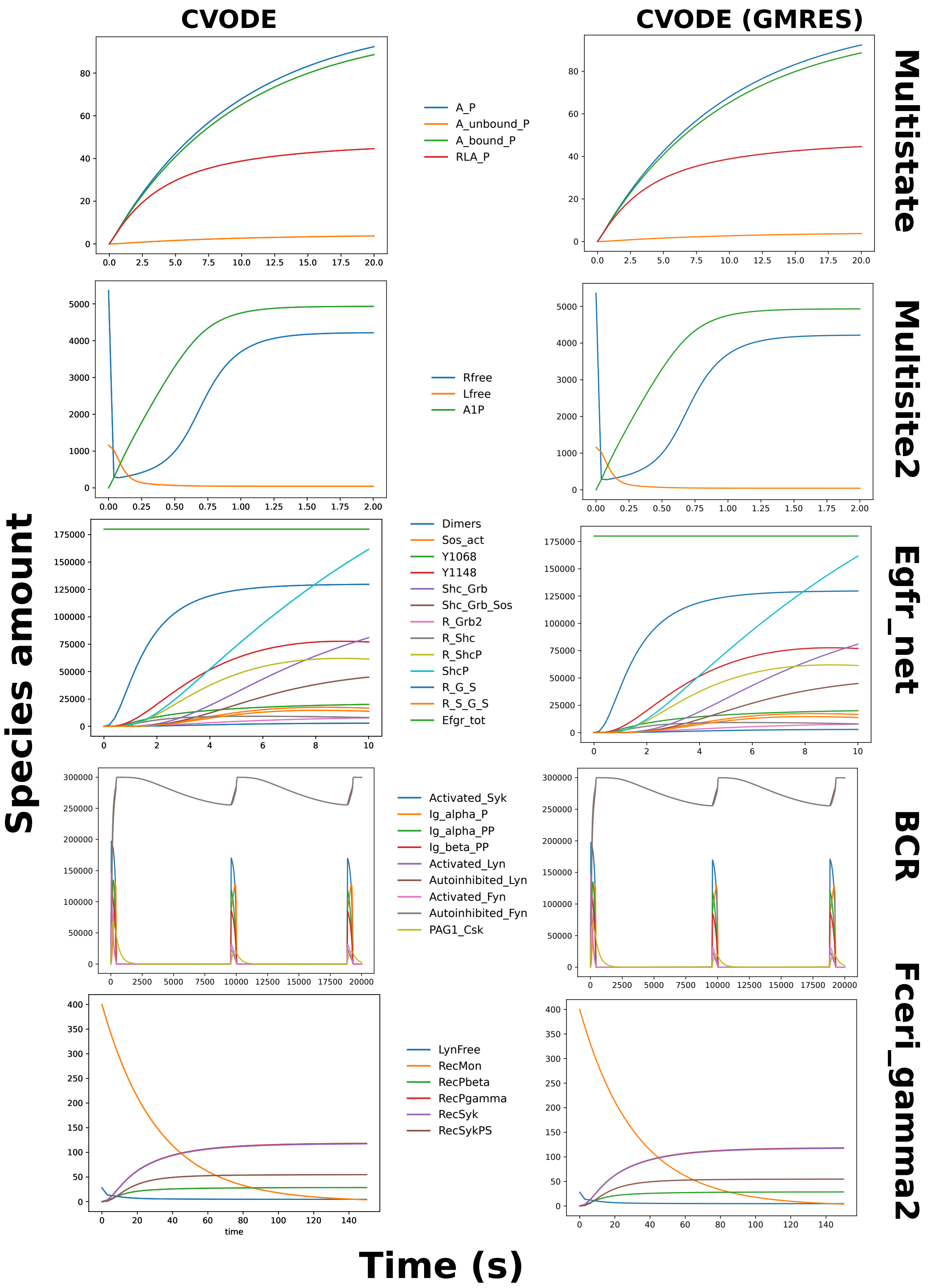

Fig I. BioNetGen benchmark simulation time trajectories. All models are simulated until they reach their (approximate) steady state (same time point which was used for the benchmarks in Fig 3). They were simulated for the CVODE method both with and without the GMRES linear solver. The simulation trajectories correspond to those of the other tools, suggesting that the models are correctly interpreted.

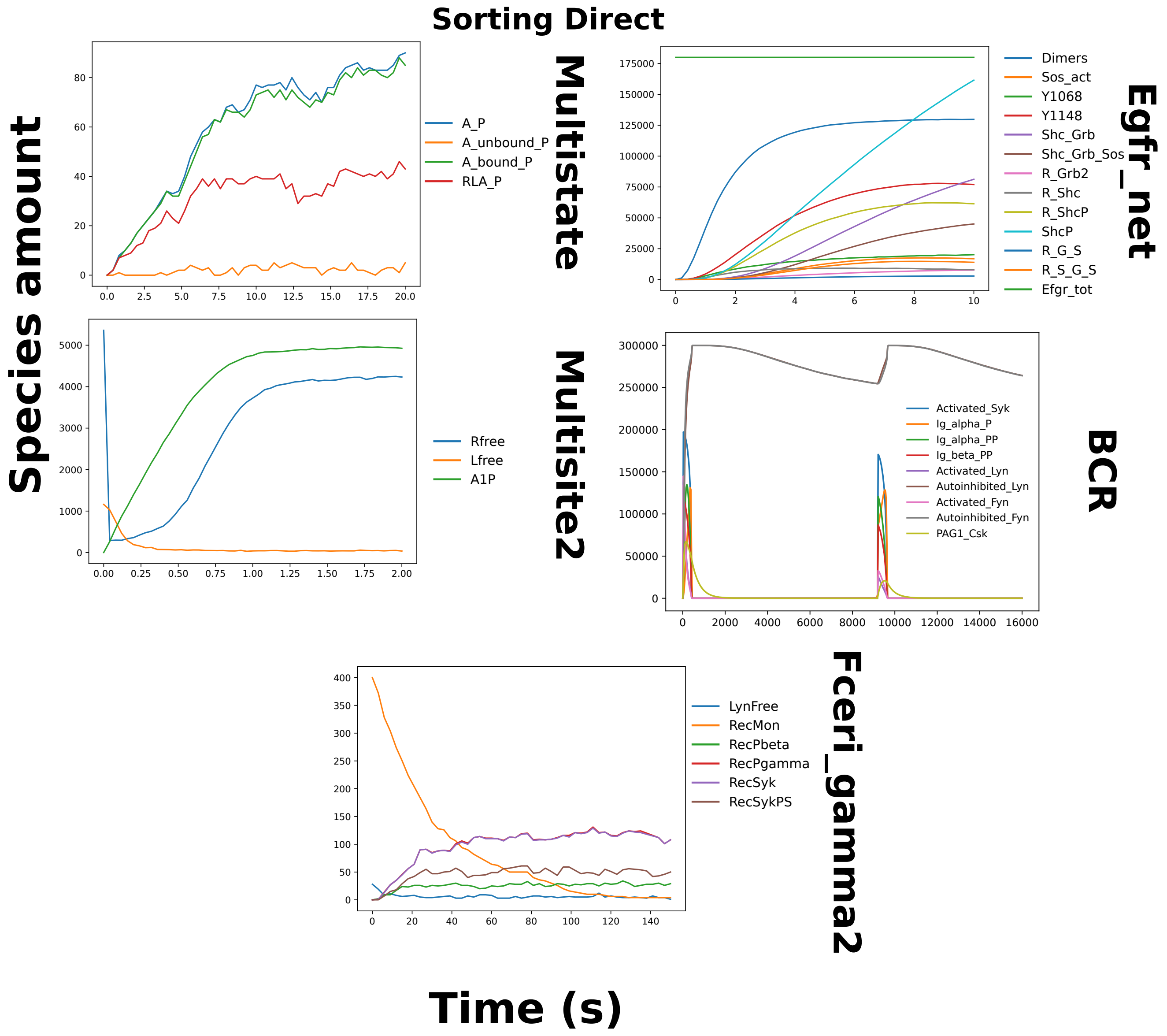

Fig J. BioNetGen benchmark simulation time trajectories. All models are simulated until they reach their (approximate) steady state (same time point which was used for the benchmarks in Fig 3). They were simulated using the Sorting Direct method. Due to the long simulation time, we did not produce trajectories for the BCR model. The simulation trajectories correspond to those of the other tools, suggesting that the models are correctly interpreted. Note that the timescale is different for the ODE and SSA simulations.

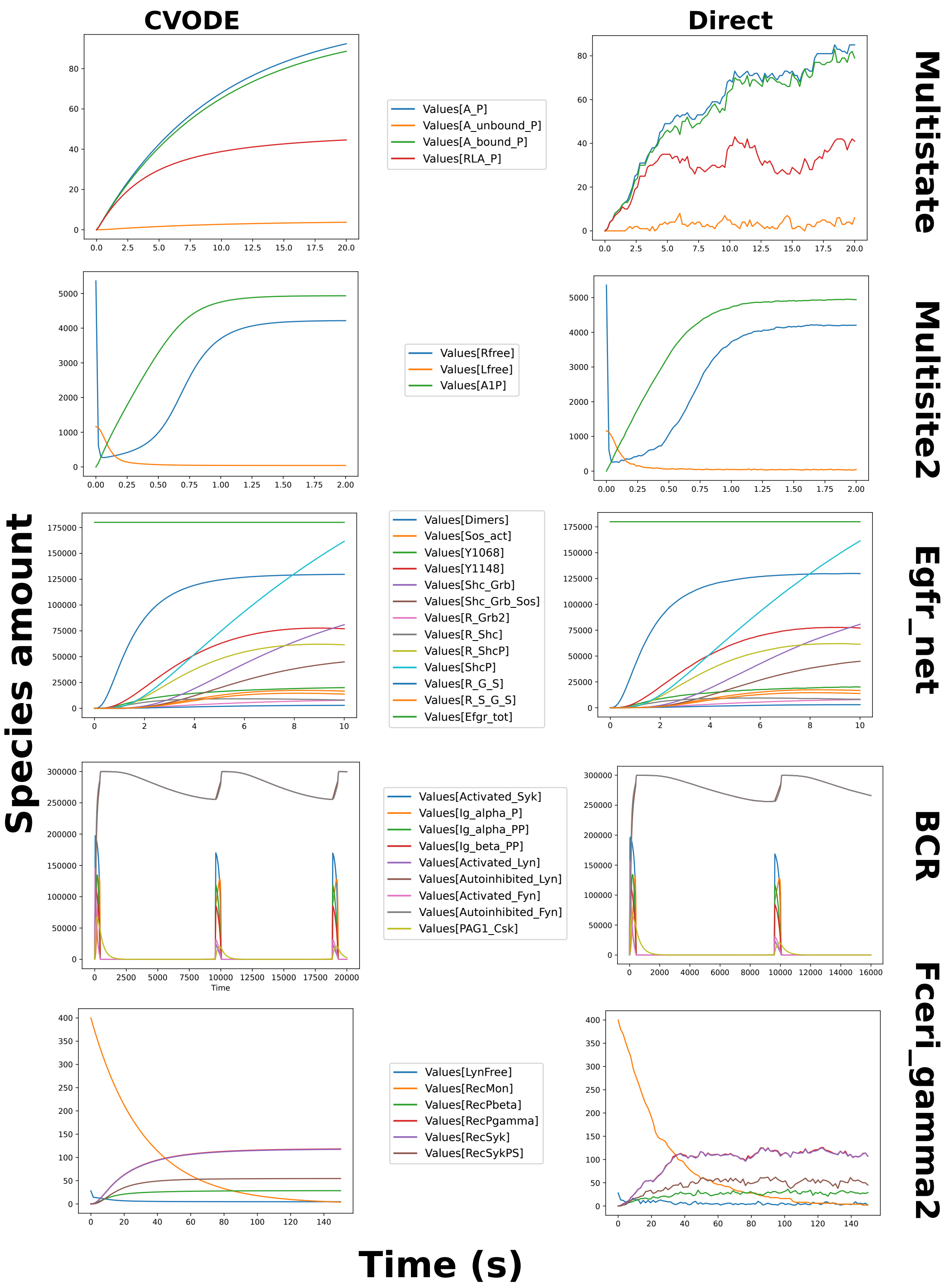

Fig K. COPASI benchmark simulation time trajectories. All models are simulated until they reach their (approximate) steady state (same time point which was used for the benchmarks in Fig 3). The simulation trajectories correspond to those of the other tools, suggesting that the models are correctly interpreted.

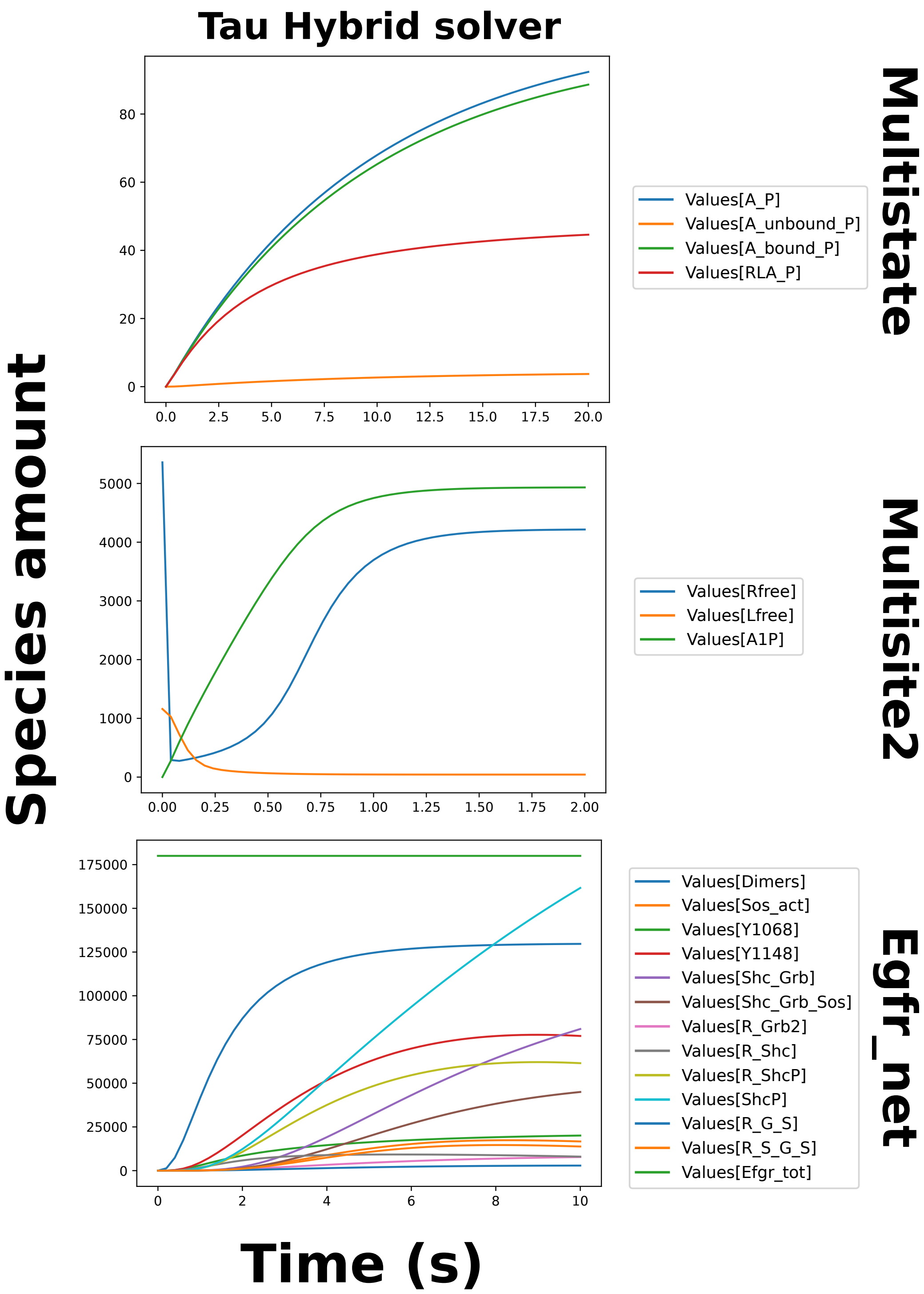

Fig L. GillesPy2 benchmark simulation time trajectories. All models are simulated until they reach their (approximate) steady state (same time point which was used for the benchmarks in Fig 3). At the time of investigation, GillesPy2 only permitted the plotting of observables when the Tau hybrid solver was used for simulation. Hence, trajectories could only be checked for this algorithm. Due to the long simulation time required for this method, we were unable to produce trajectories for the two largest models. The simulation trajectories correspond to those of the other tools, suggesting that the models are correctly interpreted.

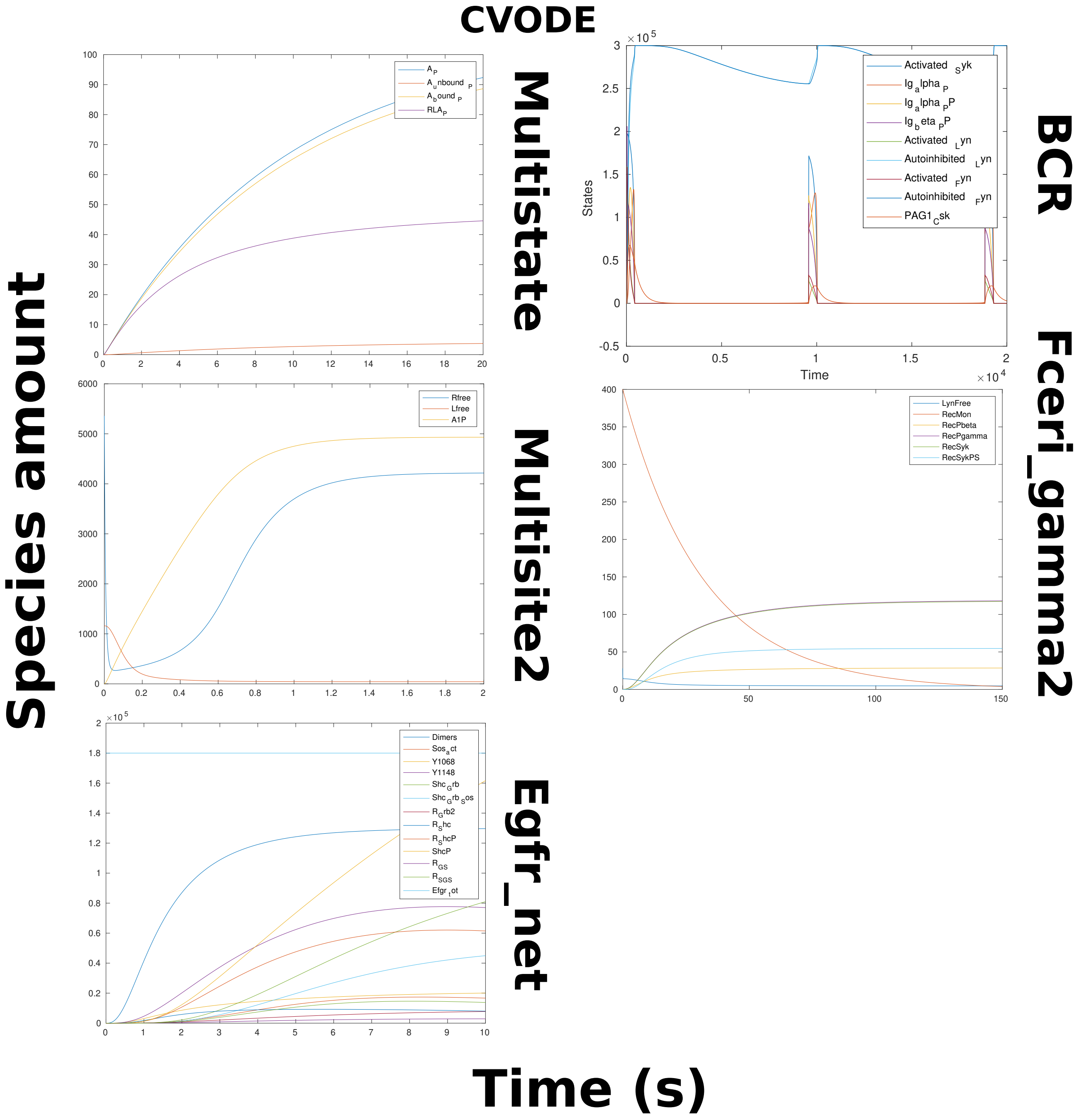

Fig M. Matlab benchmark simulation time trajectories. All models are simulated until they reach their (approximate) steady state (same time point which was used for the benchmarks in Fig 3). At the time of investigation, Matlab did not support the plotting of SBML observables from simulations using the Gillespie interpretation, hence we were unable to produce such plots. However, the ODE simulation trajectories correspond to those of the other tools, suggesting that the models are correctly interpreted. Finally, in practice, Matlab was only able to successfully complete Gillespie simulations for the smallest models.
