# Supplementary material for: Catalyst: Fast and flexible modeling of reaction networks
Source: PLoS Comput Biol. 2023 Oct 18;19(10):e1011530. doi: 10.1371/journal.pcbi.1011530 (PMC10584191; doi:10.1371/journal.pcbi.1011530)
Supplement: Attachment — Submitted filename: Catalyst_reply_to_reviewers.pdf [file pcbi.1011530.s002.pdf]

Catalyst Article, Reply to Reviewers

Reviewer 1

In the manuscript, Loman et al. present Catalyst.jl, a Julia package for modelling and simulation of chemical reaction networks. They describe how Catalyst is integrated into the SciML package ecosystem, leveraging other packages for symbolic model representation and numerical simulations. The authors demonstrate how chemical reaction networks in Catalyst can be generated, simulated, and extended or analysed further using intrinsic Catalyst features, other SciML tools and third-party Julia packages. They also perform an extensive set of model simulation benchmarks using a variety of ODE and SSA solvers, comparing Catalyst's runtime performance to that of several other popular modelling packages.

In my opinion Catalyst.jl is a great library that works beautifully in tandem with the broader Julia ecosystem for numerical simulation and higher-level analysis, making Julia the programming language of choice for many computational biologists. On GitHub, the package is well documented and includes thorough tutorials. The manuscript itself is clear to follow and does a good job in presenting Catalyst, showcasing its features and higher-level applications. I also appreciate the rigorous simulation benchmarks and their detailed descriptions.

In summary, Catalyst.jl is a great contribution to the field and I happily endorse the manuscript's publication.

Comment 1

"Data and Code Availability" in the additional info has an old URL that does not work, but the correct one is provided in the Code Availability section of the main text.

Reply: We thank the reviewer for carefully checking these links. We have double-checked each link to ensure they are all correct.

Comment 2

There appears to be a reference missing in "That a CRN can be unambiguously represented using these models forms the basis of several CRN modeling tools [?,11–22]". In addition, another software tool that perhaps should be cited given its list of features is "CERENA: ChEmical REaction Network Analyzer—A Toolbox for the Simulation and Analysis of Stochastic Chemical Kinetics", PLoS ONE 11(1): e0146732.

Reply: We thank the reviewer for pointing out this error, which we now have corrected. We have also added the reference to Kazeroonian et al. (2016).

Comment 3

In Figure 2, it might be helpful to show the Brusselator reaction diagram and assumptions made to make life easier for an unfamiliar reader, or at least mention it in the caption.

Alternatively, a more accessible reference could be useful: from a quick look at the one cited, i.e., Lefever et al. (1988), it does not seem to clearly define the Brusselator as used here and does not explicitly discuss the limit cycle condition (relationship between A and B). Perhaps "Elements of Applied Bifurcation Theory" by Y. A. Kuznetsov or another textbook would work better.

Reply: We have added this additional reference to the Brusselator (Figure 2 caption). We have also updated the figure and caption to more thoroughly describe the Brusselator CRN, show how the model is created in Catalyst, and show the underlying reactions.

Comment 4

In Section 2.2, is there a particular reason why Catalyst is benchmarked against BioNetGen, COPASI, GillesPy2, Matlab's SimBiology, and not any other packages? These seem to be chosen very reasonably given their popularity, list of features and them being actively maintained, but it might be worthwhile to mention this explicitly.

Reply: We have now updated the second paragraph in 2.2, motivating the selection of these tools (the reason being the same as suggested by the reviewer). We now say "These tools were selected as they are popular and highly cited, well documented, scriptable for running benchmark studies, and actively maintained. The Matlab SimBiology toolbox was selected due to the enduring popularity of the Matlab language. Overall, they provide a representative sample of the broader chemical reaction network modeling software ecosystem."

Comment 5

Regarding Section 2.2 and Fig. 4, it might be interesting to have even a very short discussion summarising the potential main factors leading to such notable runtime differences between CVODE and lsoda in different languages/packages, as well as almost always superior performance of native Julia solvers. Is it mostly about native language performance, specific implementations of the solvers or other algorithmic choices made?

Reply: We have added a new paragraph at the end of 2.2 mentioning some of the reasons Catalyst models might demonstrate better runtime performance. A more detailed discussion that goes beyond the scope of this manuscript, and would perhaps form the basis for a future work, follows.

The quick answer is that attributing it to any single aspect is a major over-simplification and it is a combination of many minor details. It would require an entire manuscript to clearly detail what pieces contribute to the whole picture, and without the space to share all of the evidence it becomes a subjective rather than objective comparison as to what parts matter. Therefore, we wish to restrain from making strong assertions about the specifics within the manuscript which are both subject to change over time and require many figures/plots/etc. of their own in order to justify an exact breakdown.

However, as an oversimplification to give a clearer picture to the reviewers we share the following below.

Some of the factors include that the default linear solver used in the Julia side is not a standard LAPACK implementation but instead an optimised recursive LU-factorization which tends to greatly outperform OpenBLAS (the standard BLAS shipped in most open source languages such as R and Python) and even outperform Intel's own MKL up to matrix sizes of 500x500 (see https://github.com/SciML/LinearSolve.jl/issues/357 for a more detailed comparison on many different CPU architectures as reported by users). This is in part due to improved SIMD heuristics. Given the LU factorization is one of the most expensive operations in solving with an implicit method, this performance improvement over LAPACK means that one would expect the same method to have a non-trivial performance advantage from the way it's called in Julia vs the way it's called even from the C/Fortran demos that Sundials ships with (Sundials is the C++ library that provides CVODE). In addition, it's not clear whether the other domain-specific modeling tools alter the build process to use a BLAS implementation for this aspect, as by default Sundials CVODE will build with an internal 3-loop implementation for LU-factorizations, and lsoda would require modifying the source in order to bring in an alternative factorization. So with all of the uncertainty in how this effect is dependent on the matrix size, the CPU, and the LAPACK choices of the other software, this can cause anywhere from a 50% difference to 1000% difference in performance depending on the exact scenario being looked at.

We note that such BLAS/LAPACK implementations which are not the standard CVODE/lsoda built-in will also automatically multithread LU-factorizations by default when a certain size heuristic is hit (for example, OpenBLAS's heuristic is at 200x200 matrices which is actually too small and makes OpenBLAS slower than its single threaded counterpart until around 450x450 matrices on many CPUs). The LinearSolve.jl solvers used in the Julia implicit ODE methods have a default that jumps between different implementations using known issues like this. For the purpose of these benchmarks, we used single-threaded LU-factorizations to provide a more direct comparison against the other implementations, which may not wrap a multithreaded LAPACK. Due to the aforementioned threading effect this actually improves the performance in many of the examples (that are not BCR).

"Native language performance" is an aspect that needs to be broken down into more detail. It is quite difficult to know the exact details of the differences within software here as none of the software shares examples of what the generated C code looks like from their compilation process without diving into extreme details (and in some cases this process is part of a proprietary compiler of which we would prefer to shield ourselves of any legal backlash), but we can at least note what aspects we have found to be important in such projects and speculate as to how one may see a difference. One key aspect to the code generation process is that the final code generated from Catalyst fully inlines all aspects of the rate function calculation into a single flat representation of the function. This inlining greatly improves the performance by removing small function calls. While in many cases a compiler's inlining heuristic can statically remove the function call and perform an auto-inlining, there are many edge cases to such an inlining heuristic, such as function size, that could trigger adverse behaviour and thus cause some functions to not inline. In particular, with respect to the Julia compiler Michaelis-Menton functions and Hill functions are right on the edge

according to the inlining heuristic, though faster to inline and it does inline in later Julia versions (though from memory it did not in versions before v1.3 when a new inline scheduler was added, with this function being a test case provided by me to the compiler team as something we wished to ensure inlined). A generated C code relying on GCC heuristics may lose some performance at this point, though this is exactly the kind of detail we would not want to write into a paper without doing a complete analysis proving what aspects are inlining and not inlining (and it would be a complete diversion to include a multi-page analysis of this, beyond that its usefulness is questionable since the results will be subject to change between compiler versions).

One piece to note about the Julia implementation is that the inlining of nonlinear functions is counteracted by direct optimizations for mass action terms. The generated code in many instances (such as the jumps) handles mass action terms separately from the rest of the nonlinear terms. If this was not done, the level of optimization that is applied to the general nonlinear functions would not be tenable because the code size would be too large and the compilation times would balloon (maybe at a rate of O(n^4) due to the LLVM GC Mark pass, though dependent on specifics of what must be marked and whether the compiler heuristic knows whether to remove the mark pass via complete inference). Thus mass action terms for jump problems are handled separately in a way that has been hand-optimised, looping over the mass action terms to achieve constant time compilation with respect to the number of mass action terms. Under the assumption that most terms are mass action, this is a piece that allows the compilation process to be hyper-optimising on the difficult parts while not ballooning too fast.

Another major aspect of native language performance is vectorization, vectorization not in the sense of Python/MATLAB/R but in the sense of allowing code generation to automatically determine optimal SIMD scheduling via things like SSE2 and AVX512 instructions to allow for computing multiple + and * operations simultaneously. One of the core optimizations which can be helpful here is SLP vectorization which allows for auto-vectorization of non-loop code like the generated code from inlined chemical reaction networks. Many auto-vectorization codes focus most of their effort on the automated SIMD of for loops, but in the scenario of the generated code like we see here the SLP vectorization is much more important. SLP vectorization is also much more difficult. The only documented improvements we know of to GCC completed in 2011 (https://gcc.gnu.org/projects/tree-ssa/vectorization.html). This is important because, even though GCC added a block vectorization code model which would be important in the repeated structures found in a chemical reaction network, its cost model dates back to a time where AVX512 instructions (and even some SSE2 instructions) were new enough that many CPUs would have to down-clock in order to make use of them. At a high level, this means that while a CPU could execute 4 or 8 + operations simultaneously, doing so would require an expensive instruction which would change the clock speed on your CPU temporarily in order to execute the instruction, and then require an expensive instruction to automatically bring the clock speed back to the normal rate. This means you would need to ensure that these commands are only performed if you have many of these commands, and thus cost models from this time are heavily conservative with many never using AVX512

instructions at all. More modern AMD and Intel CPUs have greatly improved the way that support for these vector instructions can be done (through improved thermal management), and thus the cost model required for more representative CPUs (and thus the SLP vectorization algorithm that would be efficient, since it's a greedy algorithm solution to an NP-hard problem) is substantially different from what one could/should/would have written in 2011. Julia's approach through LLVM uses a more modernised SLP vectorization pass (https://llvm.org/docs/Vectorizers.html) which in particular had some contributions from Intel (https://llvm.org/devmtg/2015-04/slides/MaskedIntrinsics.pdf) to ensure that this could be done.

Years ago in a much earlier Julia where this benchmarking was much more common, there were more than a few cases discussed in chat where, on toy examples (no packages, straight-line code), someone found Julia was faster than a GCC compiled C code (usually in the 2x-3x range) but matched Clang-compiled C code, where Clang is the LLVM compiler front end for C. In these cases, the difference was often attributed to differences in inlining heuristics and SLP vectorization. So while a detailed manuscript worthy of publication in a computer science outlet would be required to definitely "prove" what aspects of the compiler heuristics are likely causing a difference and to what extent they are, we can put together at a high level that (a) the kind of code Catalyst is generating can be very reliant on inlining and SLP vectorization heuristics and (b) these have been seen to be better in LLVM than GCC before, and thus hypothesise that this may be a substantial part of what could cause a difference in the generated code performance between Catalyst and the other tools which we suspect all use GCC on generated C code. Of course, this comparison would require an extreme amount of detail in order to achieve exact numbers and attributions on a per-CPU basis, and therefore we refrain from making a detailed claim in this manuscript attributing differences to this exact portion.

Another potential substantial contributor to performance differences which can be attributed to "native language performance", which comes into play with some of the smaller differential equation examples, is a detail in JIT compilation where one can embed runtime level information into the compiled function call. With ahead-of-time compilation this would be invalid since some of the runtime details may change between compilation and execution, but that's not possible in the JIT compilation setting, allowing for further optimizations. This leads to the surprising result that calling C shared libraries from C, using C's ABI, is faster from JIT compiled languages which make use of this trick than C itself (in particular Julia and LuaJIT)! See independent measurements for extra details https://github.com/dyu/ffi-overhead. This gives JIT compiled languages about a 10% performance improvement when handling shared libraries, something that would come into play with many calls to Sundials in an ODE solver loop as it interacts with the shared library for small ODEs, meaning that with all other code being equal a solver loop calling Sundials .so binaries from Julia with the same compiled ODE code would run faster in Julia than from C. The exact contribution of this effect can be quite difficult to measure in practice but is likely worth mentioning in the complete story.

One other potentially substantial effect could be the underlying math library. C defaults to using the system math library, which has different implementations on

different operating systems and thus can be difficult to attribute any single performance number to any single operating system. The Julia compiler team itself wrote and maintains OpenLibm (https://openlibm.org/) which is one of the core open source libm implementations used by some programming languages and operating systems. That said, since around Julia v1.3 all calls to OpenLibm, and thus any system math library, were removed from Julia base with all implementations being direct Julia implementations. The reason was this allowed for more rapid development and improvements of Base math functions. In particular, the differential equation solvers had their own floating point power implementation as mentioned briefly in some previous manuscripts as attributing to 2x-3x performance improvements in Runge-Kutta solvers in small ODEs due to the use of floating point power in the time adaptivity heuristics (https://www.biorxiv.org/content/10.1101/2020.11.28.402297v2). These implementations were further improved using min-max polynomials by Oscar Smith and added to Base Julia. While it seems the 64-bit version is a bit obscured in its exact performance difference, https://github.com/JuliaLang/julia/pull/40236 notes that 32-bit floats achieve a performance improvement of 1.5x in the 0.5 ulp implementation against a previous Julia-based one (which already outperformed OpenLibm), which gives a rough estimate back in that range of around 2x. We note that floating point power calculations are on the order of 100-1000 times more expensive than * and + operations, and thus chemical reaction network simulations with many Hill function terms can have performance limited by the ^ operation if the operand does not type the power as integer. A near future optimization to the ModelingToolkit code generation process used by Catalyst will allow for the operands to be correctly integral typed (https://github.com/SciML/ModelingToolkit.jl/pull/2231), thus allowing the compiler to specialize a^2 -> a*a and fully remove this effect, though with the optimizations already made in Julia we suspect that this exact effect in Hill functions is likely not too large anymore. However, we point this out as a place where specialised optimizations would be required by the other tools. Some SBML models treat this operand as an integer literal, in which case the code generation process with GCC and standard system libraries would need to avoid naively lowering to a pow/powf call and directly perform this operation in the code generation process in order to not see a slowdown. And in some cases SBML models treat this value as an integer parameter, and thus if the lowering is done to a single parameter vector of floats this would lead to a pow/powf call as well, and therefore the lowering would need to manually split these coefficients out to avoid the system library call. This is deep in the code generation pipeline and thus it is very difficult for us to determine exactly how other programs are doing this lowering (and impossible in some cases that are proprietary), but given the effect we've seen over the years of optimising this effect we can at least point out that it would require manual intervention that we suspect is not being done.

In conclusion, it depends. We hope the reviewers are interested in such a high-level heuristic breakdown but at the same time understand that the amount of effort to really nail down all of these details would be a manuscript itself (and a moving target as CPU architectures and compiler infrastructure changes). This insight comes from years of developer chats with the compiler teams and users and any overly succinct summary would require a significant amount of work to produce the sufficient

evidence included with the manuscript to give direct attributions to the level of contribution from each effect. Though knowing these effects, we hope the reviewers understand why such a performance should be expected a priori.

Comment 6

In Section 2.4, it might be more fitting with the paragraph style to specify which Julia packages allow approximating the unknown CRN structures using neural networks.

Reply: We have now clarified this functionality is enabled by the SciMLSensitivity package in Section 2.4 where we now write: "Furthermore, unknown CRN structures (such as a species's production rate) can be approximated using neural networks and then fitted to data."... "This functionality is enabled by the SciMLSensitivity package [60]."

Comment 7

Would be interesting to hear more details about the planned updates for spatial model support mentioned in the Discussion. Would that be a compartmentalised approach (akin to reaction–diffusion master equation) and what specific spatial SSA solvers would it include? Or does this also imply support for continuous reaction-diffusion processes?

Reply: We currently have several work in progress PRs, see the Catalyst.jl repo, on adding spatial support for graph-type models (where transport is between nodes of a graph). For jump process models this would indeed encompass the reaction-diffusion master equation, though our longer-term goal is to support non-local reaction structures too and allow the use of the convergent reaction-diffusion master equation and other classes of models. JumpProcesses.jl currently has two RDME solvers, though both are currently limited to only mass action type reactions. One is based on the next subvolume method and the other, more performant, method uses the composition-rejection direct method to select the site of the next transition, and then uses the direct method to choose the event that occurs at that site.

On the ODE side these PRs should allow support for a variety of spatially-discretized PDE models, where the discretized transport operator can be represented as transitions within a graph. They can also be used for modelling transport in models involving discrete compartments. Ultimately, we hope to also allow continuous-space reaction network representations, and leverage other packages like MethodOfLines.jl to automatically discretize the associated reaction-diffusion PDE models and/or generate spatial transition matrices for spatial SSAs. Should suitable Brownian Dynamics or other particle-type solvers become available as Julia libraries, we would certainly be interested in interfacing with them too via such a continuous-space reaction-network representation.

We have updated the discussion to now say "This includes specialised support for spatial models, including spatial SSA solvers for the reaction-diffusion master equation, and general support for reaction models with transport on graphs at both the ODE and jump process level. A longer-term goal is to enable the specification of continuous-space reaction models with transport, and interface with Julia partial

differential equation libraries to seamlessly generate such spatially-discrete ODE and jump process models."

Comment 8

One quality of life improvement in Catalyst would be allowing to associate a volume parameter with each compartment and hence automatically scale the mass-action reaction rates according to volume. On a different note, an interactive GUI even in a very limited form (tutorials/specific Pluto notebooks?) could make the software more appealing for biologists with little to no programming experience. I see that both these comments are to some extent covered in the issues on GitHub, but perhaps expanding on future work and touching upon these and other similar possible improvements would be an insightful addition.

Reply: We thank the reviewer for these suggestions, and hope they will continue contributing any ideas or suggestions they have, using GitHub or other means. While volumes are currently supported, in the sense that a user can define a volume parameter and include it in rate expressions, it is true that we do not provide automatic conversion from concentration units to "number of" units (or vice-versa). This is certainly something that has been requested by several users and is in our thoughts, though we haven't yet figured out how we would like to handle this / what the interface should be (suggestions are welcome!). We have added the following sentence to the discussion: "Finally, given Catalyst's support for units we hope to implement functionality for automatically converting between concentration and "number of" units within system specifications by allowing users to specify compartments with associated size units."

A complete GUI for acausal modeling which integrates Catalyst for chemical reaction network components, known as JuliaSim, is being developed by JuliaHub. This is a separate project which is proprietary of which authors Chris Rackauckas and Yingbo Ma are associated (though free for academic and non-commerical usage). To avoid confusion we leave out any mention of this in the current manuscript and focus strictly on the open source Catalyst library (and therefore the model representation and code generation). We note that ModelingToolkit, Catalyst, and all of SciML are fully free and open source (MIT licensed) and thus we encourage any third party to build GUI components off of Catalyst as they see fit, and have seen some small scale third party projects already doing this (for example see https://github.com/bradcarman/ModelingToolkitDesigner.jl).

Adding some example interactive tutorials via Pluto or Jupyter notebooks to illustrate how users can easily get basic interactive environments is definitely something we will consider (and we have opened an issue on this as a future work).

Reviewer 2

In the manuscript entitled 'Catalyst: Fast and flexible modeling of reaction networks' by Loman et al the authors developed a tool, Catalyst.jl, to describe biochemical reaction networks in the framework of Julia programming language. The Julia library Catalyst.jl is a symbolic modelling package where users can create the network model using Catalyst's domain specific language (DSL). The models created using this framework can now be

simulated to generate deterministic or stochastic trajectories using various types of methods available in the existing package in Julia (DifferentialEquations.jl). Furthermore models developed in the Catalyst can also be used for variety of other purposes (e.g. bifurcation analysis, parameter estimation) using existing tools under Julia library.

There are many types of standalone modelling tools (e.g. COPASI, BioNetGen etc.) available to systems biology researchers for simulating biochemical reaction networks. The main appealing factor of Catalyst, in my opinion, is that in can be integrated into diverse types of existing Julia programming tools to achieve the desired objective. I recommend publication of the manuscript in the Plos Computational Biology upon justification of the following points.

Comment 1

Often system biology models are phenomenological in nature where the reaction rates are nonlinear with phenomenological rate functions. Although the authors mentioned about Hill function, however it is not clear whether user can customize the rate function as needed.

Reply: We apologise for any confusion caused by our wording. Indeed, any general Julia function of species amounts, parameters, and time, is allowed for defining a reaction rate. We have now clarified this in the text in Section 2.1 where we now say "Each reaction rate can either be a constant, a parameter, or a function. Predefined Michaelis–Menten and Hill functions are provided by Catalyst, but any user-defined Julia function can be used to define a rate."

Comment 2

Does Catalyst allow non-integer Hill coefficient?

Reply: Yes, non-integer Hill coefficients can be used.

Comment 3

Due to the gaussian nature of the noise, solution of chemical Langevin equation may lead to negative concentration/population if the copy number of the relevant species becomes very low. How this scenario is addressed in the Catalyst?

Reply: We currently wrap the rate laws within square roots that represent noise strength in absolute values, preventing square roots of negative numbers, following the approach of Higham (ref. [68]). This allows SDE solvers to still time step even when populations become negative, with a behaviour for the noise terms which is approximately reflective around the 0. This should be made a user-selectable option though, as it may be preferable for users to receive an error message about complex numbers arising in the solution process (and we have opened an issue to add such flexibility and better document / illustrate this issue and what users can do in our tutorials). Using this form of reflection allows users to be able to identify when such a case occurs in a post-processing via analysing whether any concentrations are negative, and either manually post-process states via absolute value or manually error.

More generally though, a model where such issues arise with the CLE suggests the CLE is not the appropriate physical representation to use for the system in the desired parameter regimes (at least beyond the first time a species population becomes zero and the CLE therefore becomes undefined) and any non-erroring CLE simulation would only be an approximation. A better approach would be to allow hybrid models that can dynamically shuffle species and reactions between different physical representations (i.e. jump process, tau-leaping, CLE, or ODE) as appropriate. Such hybrid models are not currently supported, but as mentioned in the discussion, are a future goal.

We have modified the discussion to now say: "Such hybrid approaches can help to overcome the potential negativity of solutions that can arise in τ-leaping and CLE-based models [67]. In the CLE case, Catalyst currently wraps rate laws within the coefficients of noise terms in absolute values to avoid square roots of negative numbers, allowing SDE solvers to continue time-stepping even when solutions become negative (following the approach in [68]). We hope to also integrate alternative modelling approaches, such as the constrained CLE [67], which avoid negativity of solutions via modification of the dynamics at the positive-negative population boundary."

Comment 4

Is the computational efficiency due to the Catalyst or the differential equation solvers developed in the Julia language?

Reply: Please see the extended response to Comment 5 of the first reviewer. It is likely due to both features provided by Catalyst (inlining mass action rate laws within the generated ODE derivative function, analysing and binning jumps into the most performant representation supported by JumpProcesses.jl, etc), coupled with the broad variety and extensive features of the differential equation and jump process solvers in DifferentialEquations.jl and JumpProcesses.jl. We have added a paragraph at the end of Section 2.2 discussing this.

Other changes

We have taken the opportunity to add references and comments on additional CRN modelling tools in the list of references within the introduction. We have also added an "Author summary" section, as required for PLOS Computational Biology articles.
